# Multinomial logistic regression analysis of the determinants of anaemia severity among children aged 6–59 months in Ghana: new evidence from the 2019 Malaria Indicator Survey

**DOI:** 10.1186/s12887-023-03919-0

**Published:** 2023-02-27

**Authors:** Desmond Klu, Donatus Yaw Atiglo, Aaron Kobina Christian

**Affiliations:** 1 Institute of Health Research, University of Health and Allied Sciences, Ho, Ghana; 2 Regional Institute for Population Studies, University of Ghana, Legon-Accra, Ghana

**Keywords:** Anaemia, Malaria Indicator Survey, Determinants, Children, Ghana

## Abstract

**Background:**

Anaemia among children under age five is a major public health issue. Although anaemia prevalence is declining in Ghana, the severity among anaemic children is worsening. This study aims to investigate the determinants of anaemia severity among children aged 6 to 59 months in Ghana.

**Method:**

The study utilized a weighted sample of 1,258 children with anaemia with data obtained from the 2019 Ghana Malaria Indicator Survey. The predictor variables included maternal, household child and health system characteristics. SPSS version. At the multivariate level, three different multinomial logistic models were run with selected predictor variables. All tests were conducted at the 95% confidence level.

**Results:**

The overall anaemia prevalence among children under age five was 43.5%. Of these, 2.6% were severely anaemic, 48.5% were moderately anaemic, and 48.9% had mild anaemia. The multinomial analysis showed that maternal, household, child and health system factors significantly predicted anaemia levels among anaemic children. The results indicate that a lower likelihood of anaemia severity is likely to be found among children whose mothers belong to Pentecostal/Charismatic faith (AOR = 0.18-model I; AOR = 0.15-model III) and children who tested negative for malaria (AOR = 0.28-model II and III). Again, a higher probability of anaemia severity was found among anaemic children whose mothers were not aware of NHIS coverage of malaria (AOR = 2.41-model II, AOR = 2.60-model III). With regard to moderate anaemia level, children who belong to the poorest, poorer and middle household wealth index had a higher likelihood of being moderately anaemic compared to those in rich households. Similarly, anaemic children who were less than 12 months old (AOR = 2.21-model II, AOR = 2.29-model III) and those between the ages of 1–2 years (AOR = 1.84-model II, AOR = 1.83-model III) were more likely to have moderate anaemia levels.

**Conclusion:**

The study findings show the importance of understanding the interrelation among different factors that influence anaemia severity among children under age five as critical in developing strategies and programmes aimed at addressing childhood anaemia.

## Introduction

Anaemia is referred to as a condition in which the level of haemoglobin (Hb) in the body is below normal, leading to a reduction in the capacity of red blood cells to carry oxygen to body tissues [1, 2]. Children are most affected, even though anaemia affects all population groups. The World Health Organization (WHO) reported that the global prevalence of anaemia in children was 60.2% in 2019 [3].

According to the WHO guidelines, anaemia in children aged under 5 years is defined as a haemoglobin concentration < 110 g/L [2]. Anaemia levels are of three types, namely, mild, moderate and severe. Mild anaemia is associated with haemoglobin concentration levels in the interval of 10.0–10.9 g/dL, moderate 7.0–9.9 g/dL, and severe less than 7.0 g/dL [4]. Iron deficiency is considered the most common cause of anaemia; other causes include acute and chronic infections that result in inflammation and blood loss, deficiencies in other vitamins and minerals, especially folate, vitamin B12 and vitamin A, and genetically inherited traits, such as thalassaemia [4–6]. Other conditions, such as malaria, genetic disorders, and cancer, also play critical roles in anaemia [2, 4].

Anaemia is often associated with increased risks for maternal and child mortality, especially in sub-Saharan Africa. Anaemia is estimated to have been responsible for 5% to 18% of underfive deaths in Africa [5, 6]. Additionally, in the African region, an estimated 3.3% of children aged 6–59 months suffer from severe anaemia, and this estimate is twice the global prevalence rate [3]. Iron-deficiency anaemia negatively affects the cognitive and physical development of children [7, 8] and results in symptoms such as fatigue, weakness, dizziness, heart failure and shortness of breath [9].

In Ghana, the national prevalence of anaemia among children aged 6–59 months has decreased over the years. For instance, childhood anaemia decreased from 75% in 2003 to 66% in 2014 according to the Ghana Demographic and Health Surveys (GDHS). More recently, childhood anaemia has further decreased from 52.7% in 2016 to 42.5% in 2019 according to the 2016 and 2019 Ghana Malaria Indicator Surveys (GMIS) report. These reductions in childhood anaemia prevalence could be attributed to malaria-related interventions, which have been associated with a 60% reduction in the risk of anaemia [10]. Despite the reduction in childhood anaemia, the levels of anaemia (severity) increased from 1.9% in 2016 to 2.6% in 2019 [10]. This calls into question the effective management of childhood anaemia in Ghana.

Using nationally representative malaria data (GMIS) provides a unique advantage over the other demographic and health survey (GDHS) because the GMIS specifically and in detail collected data on ownership and use of mosquito bed nets, assessed coverage of intermittent preventive treatment to protect pregnant women against malaria, identified practices and specific medications used to treat malaria, measured indicators of malaria knowledge and communication messages, and estimated the prevalence of malaria and anaemia among children aged 6–59 months. This gives more accurate and robust results and reflects the malaria and anaemia situation among vulnerable populations, such as children under age five in Ghana.

Several studies using nationally representative [11–15], health facility-based [16–20], school- and community-based cross-sectional data [21–26] have examined demographic, social, economic, household, nutritional, environmental, health system and spatial/geographical factors predicting childhood anaemia status.

Although evidence from the literature reveals that anaemia levels among children have multifactorial causes that negatively affect child health, the combined effect among these factors at varied levels is understudied among anaemic children aged 6–59 months. As a result, the combined effects of maternal, household, child and health system-related factors predicting anaemia levels among anaemic children under the age of five are not well understood. This study, therefore, examined the combined effects of multiple related factors (maternal, household, child and health system) that predict anaemia severity among anaemic children aged 6–59 months in Ghana using evidence from the 2019 GMIS.

## Methods

Data for this study were obtained from the nationally representative 2019 Ghana GMIS, which was conducted from September 25 to November 24, 2019. We used data from the children’s file. The GMIS collects information on malaria prevention (ownership and use of treated mosquito bed nets, coverage of intermittent preventive treatment to protect pregnant women against malaria), anaemia levels in pregnant women and children, malaria treatment and prevalence in Ghana. In this study, data on a weighted subsample of children who were tested and verified to be anaemic were extracted and analysed.

### Study setting

Ghana is a West African country that shares boundaries with Burkina Faso to the north, the Gulf of Guinea to the south, Togo to the east, and La Cote d'Ivoire to the west. It has 16 administrative regions with a population of 30.8 million as of the 2021 Population and Housing census [27]. Accra is the capital of Ghana. Over the years, numerous interventions have been implemented in Ghana to combat anaemia, such as iron supplementation, food fortification, public education and sensitization, deworming, and parasitic infection management and prevention, especially among children under five years of age [28, 29].

### Survey and study participants

Details concerning the scope and methodology of the GMIS have already been published [10]. The GMIS is a nationally representative survey conducted by the Ghana Statistical Service (GSS), Ministry of Health (MOH) and National Malaria Control Programme (NMCP) of the Ghana Health Service with technical support from the Inner-City Fund (ICF) through the Demographic and Health Surveys (DHS) Program. The data collection was performed in two phases. The first phase comprised the household listing exercise, during which each of the 200 selected enumeration areas were visited, and information was recorded on structures. In addition, information on the names of household heads and the global positioning system (GPS) coordinates of clusters were collected. In the second phase, households and all eligible women (15–49 years) were interviewed, and children aged 6–59 months were tested for anaemia and malaria with consent from guardians or parents.

With regard to the determination of the anaemia level among children under age five, a single-use retractable, spring loaded, sterile lancet was used for the finger or heel prick. A drop of blood from the site was then collected in a microcuvette. Haemoglobin analysis was then conducted on site with a battery-operated portable HaemoCue 201 + analyser, which produces a result in less than one minute. Anaemia test results were recorded both in the Biomarker Questionnaire and on a brochure that was left with the household members that also contained information on the causes and prevention of anaemia. Parents or guardians of children with haemoglobin levels below 8 g/dl (severe anaemia) were advised to go to a health facility and a referral letter with the haemoglobin reading to show to the health worker at the facility. Informed consent was sought from respondents before collection of blood samples for testing anaemia.

### Sampling and sample size

The total number of children aged 6–59 months in the 2019 GMIS was 2,895. However, in this study, we limited the analysis to children who were tested and confirmed to be anaemic during the survey. Thus, the weighted sample of anemic children aged 6–59 months in the 2019 GMIS was 1,258.

### Study variables

#### Outcome variable

The outcome variable for this study was anaemia levels among children aged 6–59 months. Anaemia is defined in this study as a reduced level of haemoglobin in the blood, decreases the amount of oxygen reaching the tissues and organs of the body and reduces their capacity to function. The categorization of anaemia level among children aged 6–59 months was severe, moderate, and mild.

#### Predictor variables

##### Maternal-related factors

We considered maternal, household, child, and health system-related factors in this study. The rationale for choosing these factors at different levels is that they may influence the anaemia levels differently.

Maternal-related factors comprised the age of the mother (15–29, 30–39, 40–49), educational level of the mother (no education, primary, secondary/higher), mother’s place of residence (urban, rural) and mother’s ecological zone of residence (coastal zone, middle belt, northern zone). Others are mother’s parity (1–3 children, 4–6 children, 7 or more children), religious affiliation of mother (Catholic, Protestant, Muslim, Pentecostal/Charismatic other Christian, Traditional/Spiritualist, no religion) and literacy level of mother (illiterate, literate).

### Household-related factors

We considered the following household-level factors in the study: sex of household head (male, female), age of household head (20–29,30–39, 40–49, 50–59,60–69, 70 +) and household wealth quintile (poorest, poorer, middle, richer, richest). The other variables included household source of drinking water, type of toilet facility and type of cooking fuel used by the household. The measurement and classification of the variable ‘*household source of drinking water’ and the type of toilet facility used* were guided by the WHO/United Nations International Children’s Emergency Fund Joint Monitoring Programme for Water Supply, Sanitation and Hygiene (WHO/UNICEF-JMP) classification of source of drinking water. For this study, the variable was classified into two categories: improved and unimproved sources of drinking water. In this study, the improved source of drinking water comprised pipe-borne water inside the dwelling, piped into the dwelling, pipe to yard/plot, piped to the neighbor’s house/compound, tube well water, borehole, protected dug well, protected well, protected spring and rainwater collection, bottled water and sachet water. The unimproved source of drinking water in this study included unprotected wells, surfaces from spring, unprotected springs, rivers/dam, tanker trucks and carts with small tanks. The type of toilet facility was also categorized as improved or unimproved. The improved toilet facilities in this study comprised flushing to pipe sewers, flushing to septic tanks, flushing to pit latrines, flushing to unknown places, flushing to biodigesters, ventilated improved pit latrines (VIPs), pit latrines with slabs, pit toilet latrines and composting toilets. The unimproved toilet facility included flush to somewhere else, pit without slab/open pit, no facility, bush/field and hanging toilet/latrine. The type of household cooking fuel was categorized into the following: liquefied petroleum gas (LPG), charcoal, fuel wood and other cooking fuel (straw/shrub/grass, agricultural crops, and animal dung).

#### Child-related factors

The child-related factors considered in the study were sex of child (boy, girl), current age of child (less than 12 months, 1–2 years, 3–4 years), child slept under treated bednet (no, yes, household do not have bednet), allow child to be vaccinated against malaria (no, yes) and child malaria status (child tested negative for malaria, child tested positive for malaria).

#### Health system-related factors

We considered the following health system level factors in this study: coverage by the National Health Insurance Scheme (Yes, No), number of antenatal visits (no visit, 1–3 visits, 4 + visits), took sulfadoxine-pyrimethamine (SP) to prevent malaria during pregnancy (no, yes) and awareness that malaria is covered under the NHIS (no, yes).

### Statistical analysis

The analyses of the data were performed in three stages using SPSS version 25. The first stage was the use of simple descriptive statistics to describe the outcome and predictor variables. The second stage involved a bivariate analysis or cross-tabulation of all the maternal, household, child, and health system-related factors against the anaemia level of children aged 6–59 months. In the third stage, we developed three different multinomial regression models to examine the combined effect of maternal-, household-, child-, and health system-level factors on anaemia levels among children aged 6–59 months. Model I analysed the effect of maternal- and household-related factors, and model II analysed the effect of child- and health-level factors. The last model analysed how maternal, household, child and health system-related factors combine to influence anaemia levels among children under age five in Ghana. All variables were considered statistically significant at the 95% confidence interval (*p* < *0.05*).

## Results

### Anaemia prevalence and severity among children under age five in Ghana

**Fig. 1:**
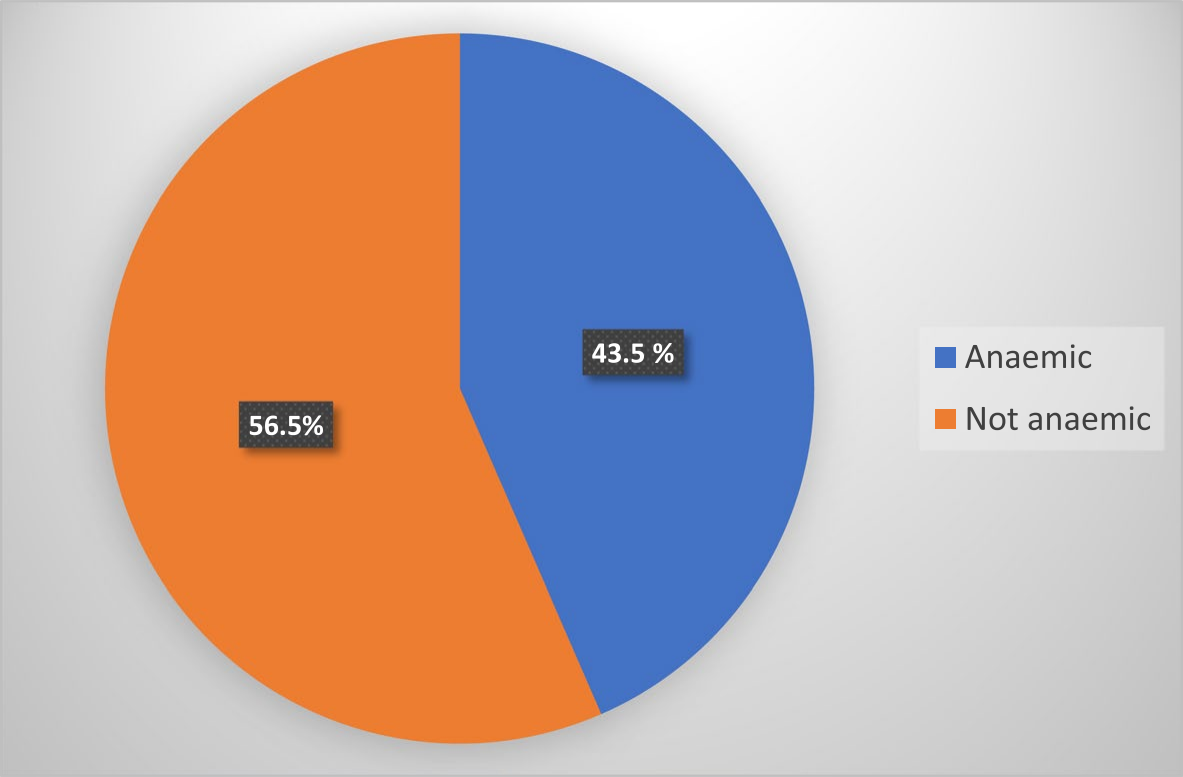
Anaemia prevalence among children under age five in Ghana

**Fig. 2:**
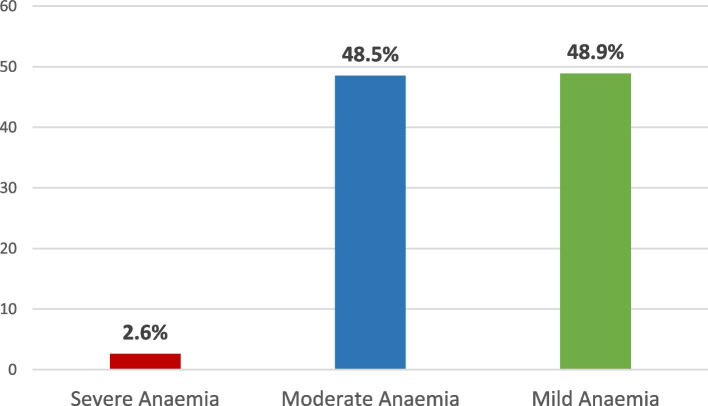
Anaemia level**s** among children under age five in Ghana

Figure 1 shows the prevalence of anaemia among children under age five in Ghana. Out of the 2,895 children aged 6–59 months, 43.5% were anaemic, while the remaining 56.5% were not anaemic. This prevalence is lower than what was found in the 2016 GMIS, with a prevalence of 52.7%. Figure 2 shows that out of 43.5% of anaemic children, 2.6% had severe anaemia, and 48.5% and 48.9% had moderate and mild anaemia, respectively. These anaemia levels are higher than those recorded in the 2016 GMIS, with 1.9% of children under age five being severely anaemic, 27.6% being moderately anaemic and 23.2% being mildly anaemic. In summary, although the prevalence of anaemia among children aged 6–59 months, as found in this study, is lower than that found in 2016, the levels of anaemia in this study are higher than those found in 2016.

### Description of predictor variables in the study

Table 1 shows the percentage distribution of maternal, household, child and health system-related factors used in this study. The highest proportion (49.5%) of mothers with anaemic children are between the ages of 15–29 years. Approximately 5 out of 10 mothers of anaemic children had attained a secondary or higher education level. Most (63.8%) mothers with anaemic children reside in rural areas, and approximately 40% of them dwell in the coastal zone of Ghana. The majority (60.3%) of mothers had 1–3 children, and a greater proportion (39.3%) belonged to the Pentecostal/Charismatic faith compared to other religious affiliations, while more than half (59.4%) of them were illiterate.

**Table 1:** Maternal, household, child and health system characteristics of respondents

Maternal related factors	Weighted Sample *n* = 1,258	%
**Age of mother**		
15–29	623	49.5
30–39	514	40.9
40–49	121	9.6
**Educational level of mother**		
No education	335	26.6
Primary	311	24.7
Secondary +	613	48.7
***Place of residence of mother***		
Urban	455	36.2
Rural	803	63.8
**Ecological zone of residence of mother**		
Coastal zone	504	40.1
Middle Belt	397	31.5
Northern zone	357	28.4
**Parity of mother**		
1–3 children	759	60.3
4–6 children	407	32.4
7 + children	92	7.3
**Religious affiliation of mother**		
Catholic	80	6.3
Protestant	135	10.7
Pentecostal/Charismatic	494	39.3
Other Christian	155	12.3
Moslem	332	26.4
Traditional/Spiritualist	25	2.0
No religion	37	2.9
**Literacy level of mothers**		
Illiterate	748	59.4
Literate	511	40.6
***Household related factors***		
**Sex of household head**		
Male	891	79.8
Female	367	29.2
**Age of household head**		
20–29	203	16.1
30–39	433	34.4
40–49	301	23.9
50–59	150	11.9
60–69	86	6.8
70 +	86	6.8
**Household source of drinking water**		
Improved water source	1075	85.4
Unimproved water source	183	14.6
**Household type of toilet facility**		
Improved toilet	748	59.4
Unimproved toilet	510	40.6
**Household type of cooking fuel**		
Liquefied Petroleum Gas (LPG)	160	12.7
Charcoal	388	30.8
Fuel wood	680	54.1
Other cooking fuel	31	2.4
**Household Wealth Index**		
Poorest	370	29.4
Poorer	295	23.5
Middle	265	21.1
Richer	201	15.9
Richest	127	10.1
***Child related factors***		
**Sex of child**		
Boy	662	52.6
Girl	596	47.4
**Current age of child**		
Less than 12 months	183	14.6
1–2 year	666	52.9
3–4 years	409	32.5
**Child sleep under treated bednet**		
Did not sleep	282	22.4
Sleep under net	811	64.4
Household do not have net	165	13.1
**Allow child to be vaccinated**		
Not allow vaccination	59	4.7
Allow vaccination	1200	95.3
**Child Malaria Status**		
Child tested negative for malaria	1131	89.9
Child tested positive for malaria	127	10.1
***Health system related factors***		
**Health Insurance coverage**		
Not covered	512	40.7
NHIS covered	746	59.3
**Number of antenatal visits during pregnancy**		
No ANC visits	287	22.8
1–3 visits	80	6.4
4 + visits	891	70.8
**Took SP during pregnancy at health facility**		
Did not take SP	348	27.7
Took SP	910	72.3
**Awareness of NHIS coverage of malaria**		
Not Aware	316	25.1
Aware	942	74.9

Source: Computed from the 2019 Ghana Malaria Indicator Survey (GMIS)

Concerning household-related factors, approximately 80% of children with anaemia belonged to male-headed households. The highest proportion (34.4%) of heads of household were between the ages of 30 and 39 years. Approximately 8 out of 10 children with anaemia belong to households that access improved sources of drinking water, while 59.4% of them belong to households that access improved toilet facilities. Additionally, 54.1% of anaemic children belong to households that use fuelwood as a main type of cooking fuel. The highest proportion (29.4%) belongs to the poorest household wealth index category. A little than half (52.6%) of children with anaemia are boys, while approximately 53%, constituting the highest proportion, are 1–2-year-olds. Approximately 6 out of 10 anemic children slept under treated bednet a night prior to the survey. The majority (95.3%) of parents or guardians indicated that they would allow their child to be vaccinated against malaria, and approximately 10% of children under age five tested positive for malaria during the survey.

Concerning health system-related factors, approximately 59% of anaemic children had health insurance coverage, while majority (70.8%) of mothers attended antenatal care four or more times during pregnancy. With regard to taking SP at a health facility during pregnancy, approximately 7 out of 10 mothers indicated taking SP, while 74.9% were aware of NHIS coverage of malaria.

### Association between maternal and household, child and health system-related factors and anaemia levels among children aged 6–59 months in Ghana

Table 2 shows the strength of association with chi-square analyses between maternal, household, child and health system-related factors and anaemia levels among children aged 6–59 months in Ghana. Maternal-related factors, including the educational level of the mother (*p* = 0.000), place of residence of the mother (*p = *0.006), ecological zone of residence of the mother (*p* = 0.000), religious affiliation of the mother (*p* = 0.001) and literacy level of the mother (*p* = 0.001), were found to be significantly associated with anaemia levels at *p* < 0.05. With regard to household-related factors, household source of drinking water (*P* = 0.001), household type of toilet facility (*p* = 0.000), household type of cooking fuel (*p* = 0.000) and household wealth index (*p* = 0.000) were significantly associated with anaemia levels among children aged 6–59 months in Ghana at *p* < 0.05. With child and health system-related factors and anaemia levels among children aged 6–59 months in Ghana. A significant association was established between current age of child (*p* = 0.000), child malaria status (*p* = 0.006), number of antenatal care visits (*p* = 0.014), uptake of SP at health facility (*p* = 0.001) and awareness of NHIS coverage of malaria (*p* = 0.047) and level of anaemia among children under age five in Ghana.

**Table 2:** Association between maternal, household, child and health system-related factors and anaemia level of children under age five in Ghana

*Factors*	*Anaemia level of Children under five years in Ghana*			
	Severe	Moderate	Mild	*P* values
***Maternal related Factors***				
***Age of mother***				
15–29	2.9	50.6	46.5	0.143
30–39	1.8	47.1	51.2	
40–49	5.0	43.8	51.2	
***Educational Level of mother***				
No Education	5.1	56.1	38.8	0.000***
Primary	1.3	48.6	50.2	
Secondary +	2.0	44.2	53.8	
**Place of residence of mother**				
Urban	1.8	43.6	54.6	0.006**
Rural	3.1	51.2	45.6	
**Ecological zone of residence of mother**				
Coastal zone	2.0	44.0	54.0	0.000***
Middle Belt	1.3	46.0	52.8	
Northern zone	5.0	57.5	37.4	
**Parity of mother**				
1–3 children	2.2	48.0	49.8	0.174
4–6 children	2.7	51.2	46.1	
7 + children	5.5	40.7	53.8	
***Religion of mother***				
Catholic	5.0	43.8	51.2	0.001**
Protestants	1.5	49.6	48.9	
Pentecostal/Charismatic	1.2	45.3	53.5	
Other Christians	3.2	44.9	51.9	
Moslem	3.9	55.1	41.0	
Traditional/Spiritualist	0.0	41.7	58.3	
No Religion	10.8	56.8	32.4	
**Literacy level of mother**				
Illiterate	3.2	52.1	44.7	0.001**
Literate	2.0	42.9	55.1	
***Household related factors***				
**Sex of household head**				
Male	2.9	49.7	47.4	0.256
Female	2.2	45.5	52.3	
**Age of household head**				
20–29	3.4	51.2	45.3	0.094
30–39	1.2	47.1	51.7	
40–49	3.7	48.5	47.8	
50–59	4.0	43.6	52.3	
60–69	0.0	50.0	50.0	
70 +	4.7	57.0	38.4	
***Source of drinking water***				
Improved	2.1	47.3	50.6	0.001**
Unimproved	6.0	54.9	39.1	
***Type of toilet facility***				
Improved	1.5	45.9	52.6	0.000***
Unimproved	4.3	52.3	43.4	
***Type of cooking fuel***				
Liquefied Petroleum Gas	0.0	38.8	61.3	0.000***
Charcoal	1.3	43.7	55.0	
Fuel wood	4.0	53.3	42.7	
Other cooking fuel	6.5	48.4	45.2	
**Household wealth index**				
Poorest	5.4	55.9	38.6	0.000***
Poorer	3.4	50.2	46.4	
Middle	1.1	48.3	50.6	
Richer	0.0	41.8	58.2	
Richest	0.0	33.9	66.1	
***Child related Factors***				
***Sex of child***				
Boy	3.0	49.8	47.1	0.323
Girl	2.2	47.0	50.8	
***Current age of child***				
Less than 12 months	1.6	57.9	40.4	0.000***
1–2 years	3.0	52.9	44.1	
3–4 years	2.4	37.2	60.4	
**Child sleep under treated bednet**				
Did not sleep	2.1	45.4	52.5	0.632
Sleep under net	2.7	49.3	48.0	
Household do not have net	3.6	49.4	47.0	
**Allow child to be vaccinated**				
Not allow vaccination	0.0	48.3	51.7	0.428
Allow vaccination	2.8	48.5	48.8	
**Child Malaria Status**				
Child tested negative for malaria	2.2	47.9	49.9	0.006**
Child tested positive for malaria	6.3	53.5	40.2	
**Health system related factors**				
Health Insurance coverage				
Not covered	2.7	48.5	48.7	0.997
NHIS covered	2.7	48.4	48.9	
**Number of antenatal visits during pregnancy**				
No ANC visits	2.4	40.4	57.1	0.014*
1–3 visits	2.5	43.2	54.3	
4 + visits	2.8	51.5	45.7	
**Took SP during pregnancy at health facility**				
Did not take SP	2.9	39.9	57.2	0.001**
Took SP	2.5	51.8	45.7	
**Awareness of NHIS coverage of malaria**				
Not Aware	4.1	51.4	44.4	0.047*
Aware	2.1	47.5	50.4	

Source: Computed from the 2019 Ghana Malaria Indicator Survey (GMIS)

^*^P < 0.05

^**^P < 0.01

^***^P < 0.001

### Combined effect of maternal, household, child and health-related factors influencing anaemia levels among anaemic children aged 6–59 months in Ghana

Table 3 shows the results of the multinomial logistics regression modelling of the combined effect of maternal, household, child and health system predictors of anaemia severity among anaemic children aged 6–59 months in Ghana. Model I examined the combined effect of selected maternal and household factors on childhood anaemia severity.

**Table 3:** Odds ratios and confidence intervals for maternal, household, child and health system factors affecting anaemia levels in children under age five (mild, moderate, and severe anaemia): Results from a multinomial logistic regression model

Factors	*Anaemia level of Children under five years in Ghana [Model I]*		*Anaemia level of Children under five years in Ghana [Model II]*		*Anaemia level of Children under five years in Ghana [Model III]*	
Maternal related factors	**Severe Anaemia**	**Moderate Anaemia**	**Severe Anaemia**	**Moderate Anaemia**	**Severe Anaemia**	**Moderate Anaemia**
*Educational Level of mother*	**Exp β [95% C.I]**	**Exp β [95% C.I]**	**Exp β [95% C.I]**	**Exp β [95% C.I]**	**Exp β [95% C.I]**	**Exp β [95% C.I]**
No Education	1.11 [0.32–3.83]	1.10 [0.73–1.68]			1.09 [0.30–3.94]	***1.19 [0.77–1.83]***
Primary	0.40 [0.11–1.52]	0.94 [0.65–1.34]			0.41 [0.10–1.65]	0.98 [0.68–1.42]
Secondary + (RC)	1.00	1.00			1.00	1.00
Place of residence of mother						
Urban	2.78 [0.97–7.93]	1.00 [0.74–1.35]			3.00 [1.00–9.04]	0.99 [0.73–1.36]
Rural (RC)	1.00	1.00			1.00	1.00
Ecological zone of residence of mother						
Coastal zone	1.11 [0.37–3.30]	0.75 [0.50–1.11]			1.09 [0.37–3.21]	0.77 [0.51–1.16]
Middle Belt	0.61 [0.18–2.06]	0.76 [0.51–1.13]			0.61 [0.18–2.10]	0.77 [0.51–1.17]
Northern zone (RC)	1.00	1.00			1.00	1.00
Religious affiliation of mother						
Catholic	0.61 [0.12–3.16]	0.59 [0.25–1.38]			0.55 [0.10–3.13]	0.45 [0.19–1.07]
Protestant	0.36 [0.06–2.29]	0.87 [0.39–1.95]			0.26 [0.04–1.83]	0.73 [0.32–1.66]
Pentecostal/Charismatic	0.18 [0.04–0.80]*	0.72 [0.34–1.51]			0.15 [0.03–0.70]*	0.60 [0.28–1.29]
Other Christian	0.50 [0.10–2.46]	0.75 [0.34–1.66]			0.42 [0.08–2.19]	0.63 [0.28–1.41]
Moslem	0.50 [0.12–2.08]	0.88 [0.41–1.88]			0.46 [0.10–2.04]	0.72 [0.33–1.56]
Traditional/Spiritualist	0.15 [0.01–3.81]	0.45 [0.15–1.34]			0.13 [0.01–3.40]	0.42 [0.14–1.27]
No religion (RC)	1.00	1.00			1.00	1.00
Literacy level of mothers						
Illiterate	0.76 [0.23–2.44]	1.11 [0.79–1.56]			0.72 [0.21–2.45]	1.14 [0.81–1.61]
Literate (RC)	1.00	1.00			1.00	1.00
*Household related factors*						
*Source of drinking water*						
Improved	0.53 [0.22–1.26]	0.81 [0.56–1.17]			0.51 [0.21–1.24]	0.82 [0.56–1.20]
Unimproved (RC)	1.00	1.00			1.00	1.00
*Type of toilet facility*						
Improved	0.76[0.33–1.71]	1.03[0.78–1.36]			0.76[0.33–1.74]	1.06[0.80–1.40]
Unimproved (RC)	1.00	1.00			1.00	1.00
*Type of cooking fuel*						
Liquefied Petroleum Gas	0.84[0.96–5.12]	1.29[0.51–3.29]			***0.49 [0.21–1.43]***	1.34[0.52–3.49]
Charcoal	0.48[0.06–3.74]	1.07[0.46–2.47]			0.41[0.05–3.26]	1.06[0.45–2.49]
Fuel wood	0.67[0.12–3.64]	1.18[0.53–2.60]			0.62 [0.11–3.46]	1.24[0.55–2.77]
Other cooking fuel (RC)	1.00	1.00			1.00	1.00
Household wealth index						
Poorest	0.27[0.10–0.64]	2.11[1.05–4.24]*			0.21[0.01–2.79]	2.36[1.15–4.84]*
Poorer	0.37[0.26. 0.75]	1.95[1.05–3.65]*			0.37[0.17–4.12]	2.20[1.16–4.18]*
Middle	0.54[0.38–0.92]	1.91[1.08–3.40]*			0.68[0.43–5.10]	2.05[1.14–3.71]*
Richer	0.52[0.46–0.98]	1.45[0.86–2.45]			0.11[0.03–3.13]	1.65[0.97–2.82]
Richest (RC)	1.00	1.00			1.00	1.00
*Child related factors*						
Current age of child						
Less than 12 months			0.89[0.22–3.60]	2.21[1.50–3.25]***	0.82[0.19–3.59]	2.29[1.53–3.42]***
1–2 years			1.56[0.66–3.66]	1.84[1.39–2.43]***	1.52[0.60–3.82]	1.83[1.38–2.44]***
3–4 years (RC)			1.00	1.00	1.00	1.00
Child Malaria Status						
Child tested negative for malaria			0.28[0.12–0.65]**	0.76[0.51–1.12]	0.28[0.11–0.69]**	0.78[0.53–1.17]
Child tested positive for malaria (RC)			1.00	1.00		1.00
Health System related factors						
Number of antenatal visits during pregnancy						
No ANC visits			0.57[0.12–2.63]	1.25[0.70–2.22]	0.29[0.06–1.49]	1.00[0.55–1.81]
1–3 visits			0.53[0.10–2.88]	0.75[0.46–1.22]	0.37[0.06–2.22]	0.62[0.37–1.02]
4 + visits (RC)			1.00	1.00	1.00	1.00
Took SP during pregnancy at health facility						
Did not take SP			1.44[0.38–5.48]	0.66[0.39–1.12]	2.07[0.50–8.67]	0.70[0.41–1.20]
Took SP (RC)			1.00	1.00	1.00	1.00
Awareness of NHIS coverage of malaria						
Not Aware			2.41[1.16–5.01]*	1.28[0.98–1.67]	2.60[1.19–5.67]*	***1.28[0.97–1.68]***
Aware (RC)			**1.00**	1.00	1.00	**1.00**

Source: Computed from the 2019 Ghana Malaria Indicator Survey (GMIS)

*RC* Reference Category

^***^p = 0.000

^**^p = 0.001

^*^p < 0.05

Mother’s religious affiliation was significant in predicting children’s anaemia level. Compared to mothers with no religion, those who belong to the Pentecostal/Charismatic faith are 82% (AOR = 0.18; C. I: 0.04–0.80) less likely to have children with severe anaemia than to have mild anaemia. There is a negative statistical relationship between the household wealth index and moderate anaemia levels among anaemic children aged 6–59 months. Anemic children who belong to the poorest (AOR = 2.11; CI: 1.05–4.24), poorer (AOR = 1.95; CI: 1.05–3.65) and middle (AOR = 1.91; CI: 1.08–3.40) household wealth index had a higher probability of being moderately anaemic compared to the richest household wealth index.

Model II examined the combined effect of child and health system factors on anaemia severity among children aged 6–59 months. Child malaria status significantly predicted childhood anaemia severity. Children who tested negative for malaria were 71% (AOR = 0.28; CI: 0.12–0.65) less likely to be severely anaemic compared to their counterparts who tested positive for malaria. Regarding health system-related factors, mothers of anaemic children who were not aware of NHIS coverage of anaemia were 2.41 times more likely to have their children severely anaemic than mothers who were aware. The current age of the child significantly predicted moderate anaemia levels among children under age five. Children who were less than 12 months old (AOR = 2.21; CI: 1.50–3.25) and between the ages of 1–2 years (AOR = 1.84; C. I: 1.39–2.43) were more likely to be moderately anaemic than those between the ages of 3–4 years.

Model III examined the combined effect of maternal, household, child and health system-related factors on anaemia severity among children aged 6–59 months in Ghana. The results show that the mother’s religious affiliation, child malaria status and awareness of NHIS coverage of malaria significantly predicted childhood anaemia severity. A lower probability of being severely anaemic was found among children whose mothers belonged to the Pentecostal/Charismatic faith (AOR = 0.15; C.: 0.03–0.70) relative to those with no religion. Children who tested negative for malaria had lower odds (AOR = 0.28; CI: 0.11–0.69) of being severely anaemic than children who tested positive for malaria. Anemic children whose mothers are unaware of NHIS coverage of malaria are more likely (AOR = 2.60; C. I: 1.19–5.67) to have their anaemia level to be severe compared to those who are aware.

Furthermore, children who belong to the poorest (AOR = 2.36; C.I:1.15–4.84), poorer (AOR = 2.20; C.I:1.16–4.18) and middle (AOR = 2.05; C. I: 1.14–3.71) household wealth index had a higher likelihood of being moderately anaemic compared to those from the richest household. Similarly, children with anaemia who were less than 12 months old (AOR = 1.72; C. I: 1.06–2.80) and between the ages of 1–2 years (AOR = 1.83; CI: 1.38–2.44) have higher odds of being moderately anaemic than children who are between the ages of 3–4 years.

## Discussion

Using the 2019 GMIS data, the study examined the independent effect of maternal, household, child and health system-related factors on anaemia levels among anaemic children aged 6–59 months in Ghana. To the best of our knowledge, this study is the first to study different related factors and how these factors affect each other to predict anaemia severity among anaemic children under age five using nationally representative data and considering the levels of severity of anaemia among children under age five in Ghana [10, 29].

The results show that 43.5% of children under the age of 6–59 months are anaemic, out of which 2.6% were severely anaemic, 48.5% were moderately anaemic and 48.9% had mild anaemia. Even though the prevalence of anaemia among children aged 6–59 months in Ghana has decreased from 52.7% in 2016 [29] to 43.5% in 2019 [10], there has been an increase in the levels of anaemia among these children. For instance, severe anaemia increased from 1.9% in 2016 to 2.6% in 2019, moderate anaemia from 27.6% to 48.5% and mild anaemia from 23.2% to 48.5%. This increasing anaemia levels among children clearly shows poor management and treatment of anaemia in Ghana. The prevalence of anaemia among children 6–59 months in Ghana is higher than the global anaemia prevalence of 39.8% in 2019 [3] but lower than the prevalence (60.2%) of anaemia in children 6–59 months in the African region in 2019 [3]. Other studies conducted within the African region found similar anaemia levels among children aged 6–59 months using nationally representative data [13–15].

The increased levels of severe, moderate and mild anaemia among children under age five, as found in this study and other studies in Africa, raise the issue of low blood haemoglobin concentrations in children as a result of iron deficiency in diets, low iron supplement intake and poor feeding practices [2, 6, 19, 30–33]. Other studies also argued that malaria plays an essential role in the aetiology of anaemia coupled with high rates of lasmodium infection in the African region increases the risk of anaemia severity, especially among children under age five [34–40].

Maternal-related factors, such as mothers’ religious affiliation, predicted the severity of anaemia among children under the age of five. Mothers who belong to the Pentecostal/charismatic faith have a lower likelihood of their children becoming severely anaemic compared to those with no religious affiliations. Previous studies conducted in Ghana [14], Tanzania [16], Ethiopia [15, 17, 18, 41] and Nigeria [26, 42] also found mothers’ religious affiliation to be a significant predictor of anaemia levels among children. Religious beliefs in food restrictions often deny mothers and their children potential dietary nutrients that have the potential to prevent anaemia. For instance, Ngimbudzi and colleagues [16] found that mothers attributed child anaemia to supernatural forces such as witchcraft or some foods (lemons, eggs, fish) eaten during pregnancy. However, after combining maternal factors with household, child and health system factors, the mother’s religious affiliation, child malaria status, household wealth index and awareness of NHIS coverage of malaria were significant in predicting the severity and moderate incidence of anaemia among anaemic children aged 6–59 months in Ghana.

This study also found that anaemic children less than 12 months old were more likely to suffer anaemia at a moderate level than children who were between 3–4 years old. Additionally, the probability of being moderately anaemic is higher among children who are between the ages of 1–2 years than among those between 3–4 years old. This implies that the risk of being anaemic is probably higher among newborns but reduces as they approach age five. This study’s findings corroborate those of earlier studies [14, 22, 23]. After controlling for other factors, the current age of the child was still significant in predicting anaemia levels among children under age five in Ghana. Furthermore, children under age five who tested negative for malaria were less likely to suffer severe anaemia before and after controlling for other variables compared to those who had malaria. This result clearly emphasizes malaria as a major risk factor for anaemia among children aged 6–59 months. Earlier studies [42–46] that examined the dichotomous relationship between malaria and anaemia among children found malaria to be a major risk factor for the development of anaemia among children.

Health system factors such as awareness of NHIS coverage of malaria at the health facility significantly predicted levels of anaemia among children aged 6–59 months in Ghana. Mothers who are not aware of NHIS coverage on malaria at the health facility are more likely to have their children suffer severe and moderate anaemia compared to those who are aware. Similar findings were reported by previous studies [47–49]. These studies reported that health insurance coverage serves as a protective factor against childhood anaemia and improves child health outcomes.

### Strengths and limitations of the study

The main strength of this study is the use of malaria-related nationally representative data to examine maternal, household, child and health system-related risk factors associated with anaemia severity among anaemic children (6–59 months) in Ghana. The results can, therefore, be generalized to all children under five at risk of anaemia infection. This study further used advanced statistical models, which accounted for the clusters within the sample.

Regardless of these outlined strengths, this study was cross-sectional; therefore, it will be difficult to deduce any causal interpretation. Finally, because the study used secondary data (children file), it could not account for other relevant risk factors, such as diet and nutrition, breastfeeding practices and iron supplement intake, which might influence anaemia severity among children under age five.

## Conclusion

Using multinomial logistic regression analysis, this study examined the combined effect of maternal, household, child and health system factors on anaemia levels among anaemic children aged 6–59 months in Ghana. This study emphasizes the essence of various factors at different levels as far as the anaemia level among anaemic children under age five is concerned. After controlling for the effect of other factors, the study established that religious affiliation (mother-related factors) has higher odds of being severely and moderately anaemic. Household-related factors, such as anaemic children belonging to the poorest, poorer and middle wealth index, had a high probability of suffering from severe anaemia. Children less than 12 months old and between 1–2 years old have a higher risk of moderate anaemia, while children 3 years old and those who tested negative for malaria have a lower risk of suffering from severe anaemia (child-related factors). Health system factors such as mothers who are unaware of NHIS coverage on malaria are more likely to have their children suffer severe anaemia. These findings suggest that in the development of strategies, policies and programmes to prevent or eradicate childhood anaemia, factors should not be considered in isolation. These maternal, household, child and health system-related factors should be considered when developing interventions to improve and strengthen anaemia prevention strategies among children aged 6–59 months.

## Data Availability

Datasets used for this study are openly available and can be accessed through https://dhsprogram.com/.

## Abbreviations

AOR: Adjusted odds ratio
CI: Confidence interval
DHS: Demographic and Health Survey
GMIS: Ghana Malaria Indicator Survey
GPS: Global Positioning System
GSS: Ghana Statistical Service
iCCM: Integrated Community Case Management
ICF: Inner City Fund
IRB: Institutional Review Board
JMP: Joint Monitoring Programme
LPG: Liquified Petroleum Gas
MOH: Ministry of Health
NMCP: National Malaria Control Programme
NHIS: National Health Insurance Scheme
SP: Sulfadoxine Pyrimethamine
SPSS: Statistical Package for Social Sciences
UNICEF: United Nations International Children Emergency Fund
WHO: World Health Organization
